# Correction: A novel 3-miRNA network regulates tumour progression in oral squamous cell carcinoma

**DOI:** 10.1186/s40364-024-00575-z

**Published:** 2024-02-27

**Authors:** Aditi Patel, Parina Patel, Dushyant Mandlik, Kaustubh Patel, Pooja Malaviya, Kaid Johar, Krishna B.S Swamy, Shanaya Patel, Vivek Tanavde

**Affiliations:** 1 Biological and Life Sciences, School of Arts and Sciences, Ahmedabad University, 380009 Ahmedabad, Gujarat India; 2 Department of Head and Neck Oncology, HCG Cancer Centre, Ahmedabad, Gujarat India; 3 Department of Cell and Molecular Biology, Iladevi Cataract and IOL Research Centre, Ahmedabad, Gujarat India; 4 Department of Zoology, BMTC and Human Genetics, School of Sciences, Gujarat University, Ahmedabad, India; 5 Bioinformatics Institute, Agency for Science Technology and Research (A*STAR), Singapore, Singapore

Biomarker Research (2023) 11:64

10.1186/s40364-023-00505-5

The original article contains a typo in the mention of miR-let7a on page 3.

The miRNA under investigation is miR-let7i as mentioned in the figure, results section and supplementary data.

