# Supplementary material for: A novel 3-miRNA network regulates tumour progression in oral squamous cell carcinoma
Source: Biomark Res. 2023 Jun 14;11:64. doi: 10.1186/s40364-023-00505-5 (PMC10268489; doi:10.1186/s40364-023-00505-5)
Supplement: Supplementary file 1 — Supplementary Material 1 [file 40364_2023_505_MOESM1_ESM.pdf]

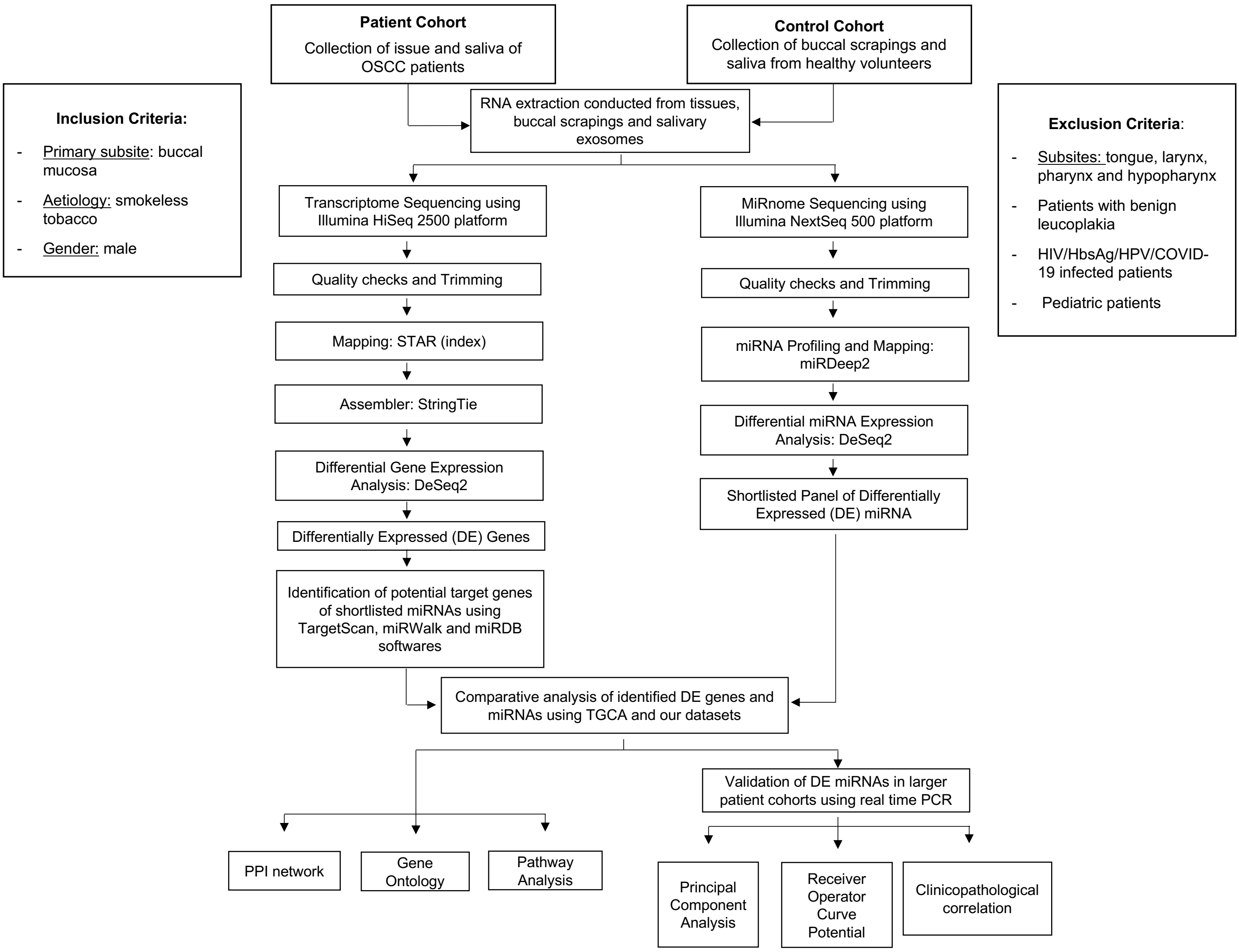

Figure S1: Overview of the study design. This figure explains the design of this study. It shows the sequencing and data analysis pipeline that was followed.

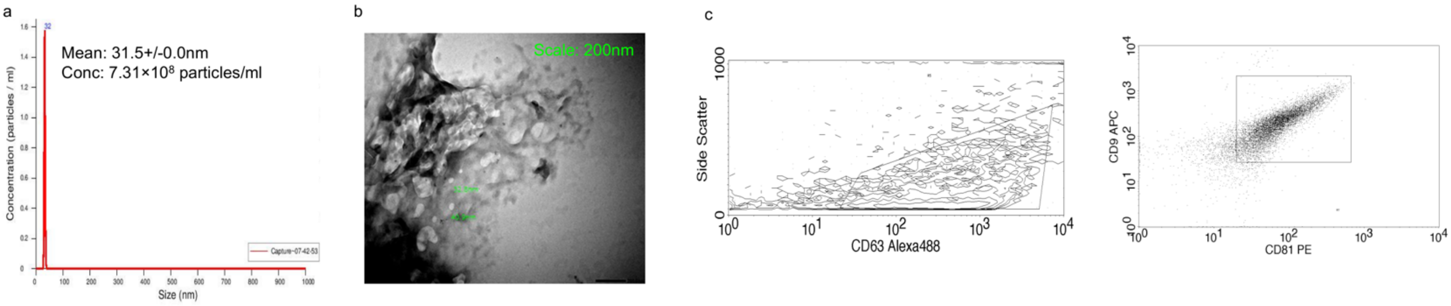

Figure S2: Characterization of salivary exosomes from OSCC patients. (a) Estimation of concentration and size of salivary exosomes derived from OSCC patients using Nanoparticle Tracking Analysis (NTA). The y-axis represents signal intensity and the x-axis indicates the size distribution of particles in NTA. **(b)** Representative TEM images of exosomes from OSCC patients, (Scale: 200 nm); **(c)** Representative plots of salivary exosomes stained with anti-CD63-Alexa fluor 488, anti-CD81-PE, and anti-CD9-APC antibodies analyzed by flow cytometry.

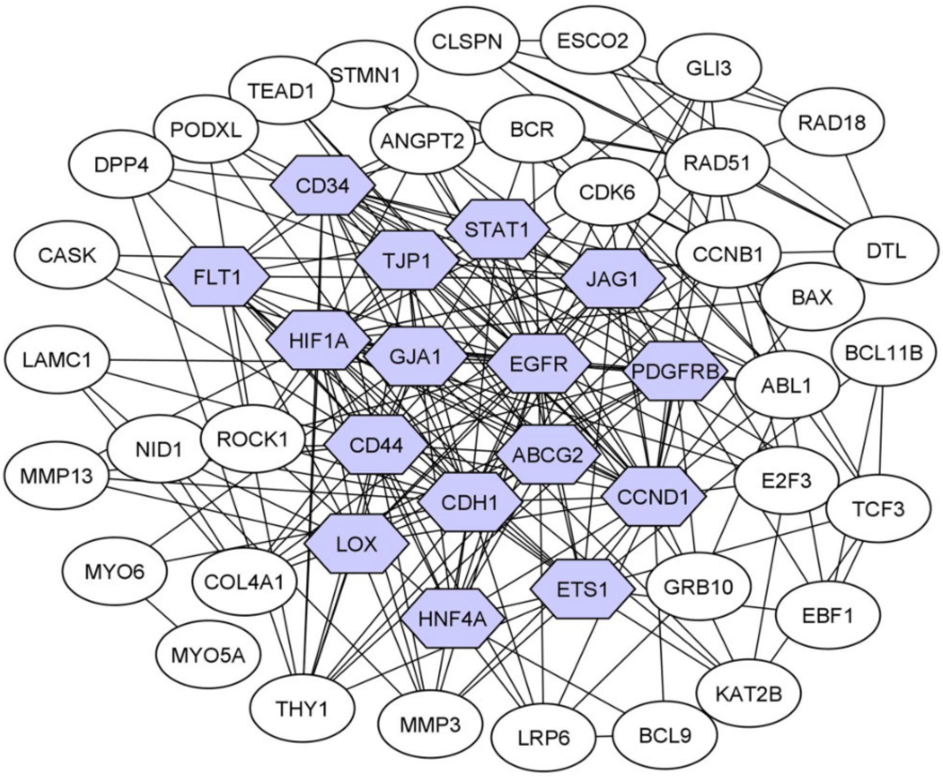

Figure S3: The gene network of mRNAs constructed using Cytoscape with Maximum Clique Centrality (MCC) Score. Protein–Protein interaction network generated in Cytoscape using STRING plugin. The hub gene analysis was performed by cytoHubba. The blue-colored nodes represent the 16 nodes with the highest MCC score.

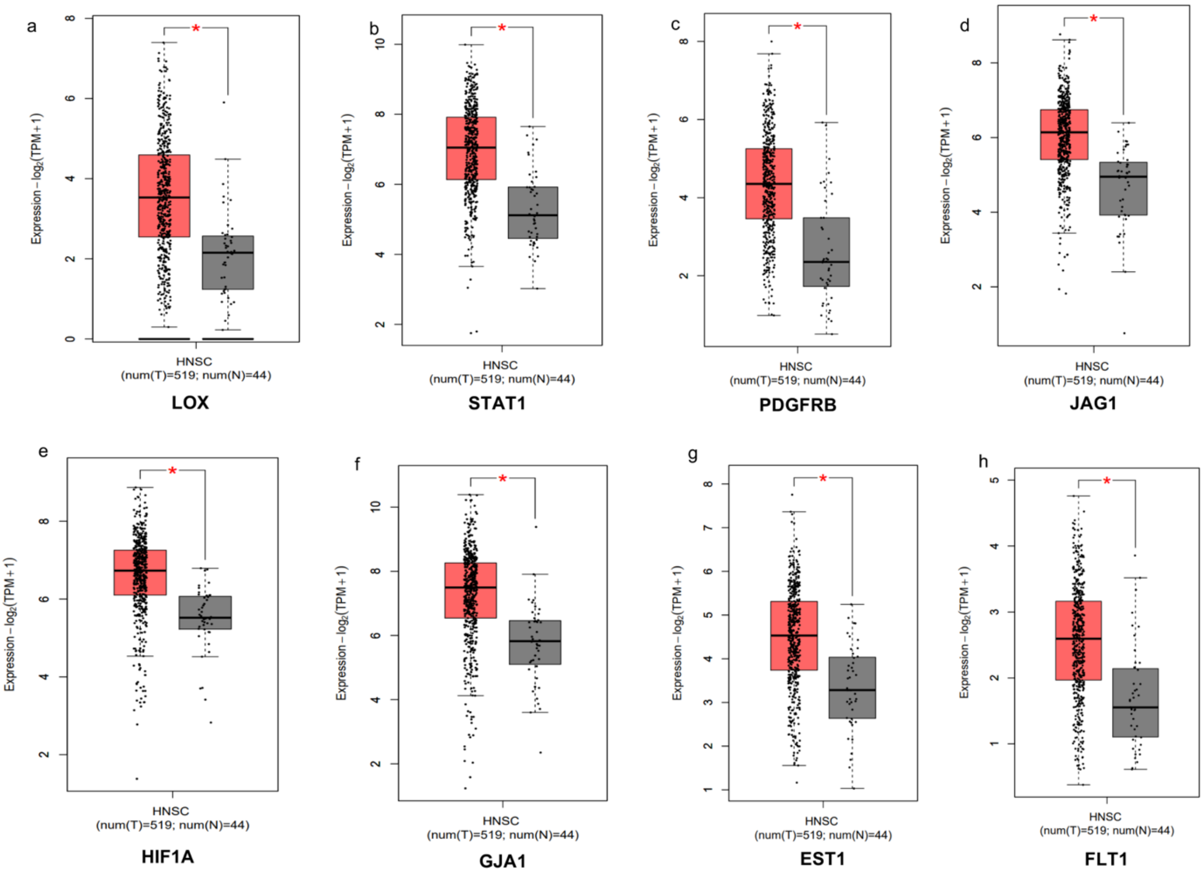

Figure S4: **Differential expression of top hub genes based on the HNSCC TCGA dataset using the GEPIA platform.** Differential expression of **(a)** LOX **(b)** STAT1 **(c)** PDGFRB **(d)** JAG1 **(e)** HIF1α **(f)** GJA1 **(g)** EST1 **(h)** FLT1 that were found to be significantly expressed in the HNSCC TCGA dataset as compared to their representative controls ($p < 0.01$), -log2(TPM+1).

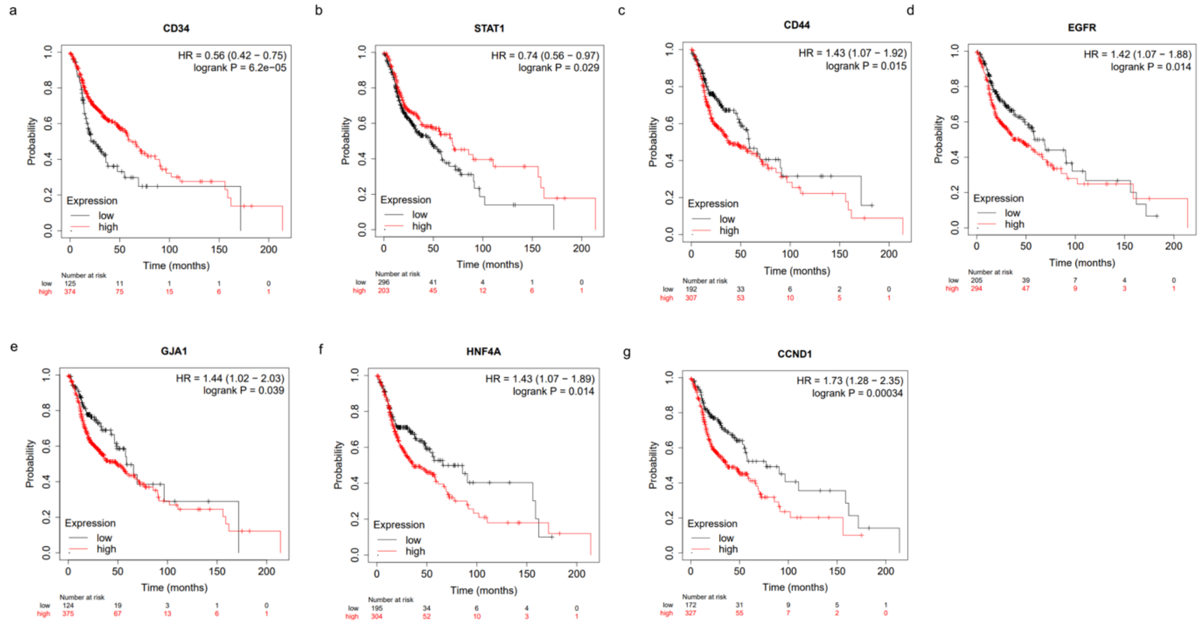

Figure S5: Overall survival analysis of hub genes performed using the GEPIA platform. Overall survival analyses performed using the GEPIA platform where patients with hub genes **(a)** CD34 **(b)** STAT1 **(c)** CD44 **(d)** EGFR **(e)** GJA1 **(f)** HNF4A **(g)** CCND1 expression above the median are indicated by red lines, and black lines indicate patients with hub gene expression below the median. Log-rank $p < 0.05$ was considered to indicate a statistically significant difference.

Table S1: **The top enriched networks generated by IPA comprising of different sets of differentially expressed genes.**

ID	Molecules in Network	Score	Focus Molecules	Top Diseases and Functions
1	ADAM9,CALU,CDH1,CITED2,CTTNBP2NL, DAB2,DLGAP1,DPP4,ERK1/2,FMNL2,GJA1, GRB10,Growthhormone,LMNB2,MMD,Mmp, MYO6,NETO2,NRIP1,NUAK1,PAXBP1,PDE4 D,PODXL,PP2A,PRKAA,PTGFR,S1PR1,SET, Shc,SPTBN1,STAT5a/b,STK38L,STRN,TJP1,T RPM7	43	28	[Cancer, Organismal Injury and Abnormalities , Respiratory Disease]
2	ABCE1,ABCG2,ACSL4,AHI1,ANKHD1/ANK HD1-EIF4EBP3, B4GALT6, CCND1, CDK4/6,CDK6,COL4A1,DTL,E2f,EGFR, estrogen receptor, G3BP1, GRHL1, Hdac,JAG1,KLF9,KLHL24,LRP6,MAP2K1/2, Mek,MGAT1,PITX2,PTPRK,RAD18,RAD51,R af,SACS,SMAD1,Smad2/3,SRGAP1,TEAD1,T HNSL1	41	27	[Cellular Response to Therapeutics, Embryonic Development, Organismal Development]
3	143,ABL1,B4GALT5,BCL11B,BCR,CAMK2G, CARD10,caspase,CD44,Creb,ETS1,FSCN1,GLI 3,LAMC1,Mapk,MMP13,MMP3,MYL12A,NFk B (complex),PDGFRB,Pka,Pka catalytic subunit,Pkc(s),PPP3CB,PRLR,RAS,RNF216,R OCK,ROCK1,RTKN,SRGAP3,ST3GAL5,TAX 1BP1,THY1,WASF1	36	25	[Cancer, Cellular Movement, Gastrointestin al Disease]
4	AARS1,Actin,BCR (complex),CCNB1,CDCA3,DGKH,E2F3,EGLN ,ELK4,ERK,FLNB,Gamma tubulin,Histone h3,HTATSF1,IgG,Jnk,KAT2B,MAP3K4,MTOR C1,NECTIN1,P-TEFb,PAN2,PDGF BB,PGM1,PPP1CC,Rb,RNA polymerase II,SLC38A2,SMAD5,STMN1,TCF3,Tgf beta,TM4SF18,TMOD3,ZNF337	28	21	[Cancer, Hematologica l Disease, Immunologic al Disease]
5	AKAP12,Akt,Alphatubulin,ANGPT2,APOL4,B 4GAT1,CD3,DLC1,DPYSL2,EEA1,F Actin,FLT1,Focal-adhesion kinase,Gsk3,HIF1A,Hsp27,Hsp70,Hsp90,KANK 2,LOX,MAP3K5,NUCKS1,P38 MAPK,PAPSS2,PI3K(complex),PI3K (family),PTGS1,RALGDS,Ras homolog,SLC4A7,SLC7A1,SRC (family),STAT1,TUBGCP3,Vegf	26	20	[Cancer, Cardiovascula r System Development and Function, Tissue Morphology]
6	BCL9,BICC1,BTF3,CDON,CSNK1D,CTNNB1 ,CUL1,CXCL14,CYP51A1,EID1,EXT1,FER,F GD4,GPR161,GPS1,HAMP,HIC1,HMG CoA synthase,HMGB3,IDI1,KIF3A,LRP4,LZTS2,M ARK1,MDFIC,NUCKS1,OSBPL1A,PLPPR4,P	17	15	[Embryonic Development, Organ Development,

	YGO1,PYGO2,RGS3,SEMA6A,SFRP4,SOST,TMEM47			Organismal Development]
7	ACP2,AHR,B4GAT1,CPM,CPSF6,DHRS9,EBF1,EFNB3,ERBB2,EXT2,FCHO2,FUT1,HECW2,HS3ST1,Igh(family),ITGB1,LMBRD2,MED1,NANOG,NUDT21,PHACTR2,PPARGC1B,S100A6,SCARA3,SEL1L3,SEMA7A,SLC43A3,SLC7A8,SLCO4A1,SMARCA4,SYVN1,TMEM106B,TPM4,TSPAN13,TUSC3	17	15	[Embryonic Development, Organ Development, Organismal Development]
8	APH1B,ATG14,BECN1,CERS6,CLSPN,CPEB3,DUT,E2F1,ERK1/2,ESCO2,ESYT1,GTPBP4,HERC2,ISCU,let7,MCM2,NAP1L1,NRBF2,OR51E1,PABPC4,PCSK5,PCSK7,PIK3R4,PSAT1,RPTOR,SNRPC,TAGLN2,THAP12,TP53,ULK2,UVRAG,VEGFD,VRK2,ZFYVE1,ZNF512B	14	13	[Carbohydrate Metabolism, Lipid Metabolism, Small Molecule Biochemistry]
9	ANGEL1,ANO6,EIF4E,EIF4EBP2,FLVCR1,HILPDA,HOXA5,HOXB5,IPO7,MXD1,MXRA5,MYC,Nc2,NELL2,NFIB,NMNAT1,NPM1,NUP50,NUP98DDX10,NUPR1,PARP1,PEG10,PLOD1,PLXND1,RPRD1B,SNHG17,SUPT5H,TCAF2,TEP1,USP36,VEGFA,YTHDC1,ZBTB34,ZFP36L1,ZNF521	14	13	[Embryonic Development, Organismal Development, Skeletal and Muscular System Development and Function]
10	ALPK3,APPL1,ARID1A,ARID4A,CEACAM6,CELSR2,CITED1,CLIC3,ESR1,FAM13C,FGFBP1,GOLM1,GPAM,GREB1,H4C3,HDAC1,HECTD1,KRT16,KRT4,KRT6A,KRT6B,MIER1,Mta,PDZK1,PIN1,PKNOX2,PRXL2A,PTGFRN,RNF181,SAP30,SLC2A4,SLC7A2,SNX24,TFF2,TOP2B	11	11	[Dermatological Diseases and Conditions, Developmental Disorder, Hereditary Disorder]

Table S2: List of top 16 hub genes with their respective interaction scores generated by the MCC method.

Gene	Score
HIF1A	75187
CDH1	74301
CD44	74210
EGFR	73498
CCND1	73068
JAG1	35528
CD34	32902
FLT1	25682
PDGFRB	22019
GJA1	16607
ETS1	15977
STAT1	15975
ABCG2	15843
HNF4A	15369
TJP1	15262
LOX	11123
PDGFRB	22019

Table S3: Primer sequences of miRNAs and genes.

miRNA	Primer Sequence
miR-140-5p	**F** 5'-GAGTGTCAGTGGTTTTACCCT-3'<br>**R** 5'GCAGGGTCCGAGGTATTC-3'
miR-143-5p	**F** 5'-GGGACAGACACCCGTTTTGA-3'<br>**R** 5' GTGTTGCCCACGGTAATGCT-3'
miR-145-5p	**F** 5'-CAGAGTGCGTGTCGTGGAGT-3'<br>**R** 5'-AGGTCCAGTTTTCCCAGG-3'
miR-30a-5p	**F** 5'-GGGCCTGTAAACATCCTCG-3'<br>**R** 5'-GAATACCTCGGACCCTGC-3'
miR-423-5p	**F** 5'-TTGGAGTAGGTCATTGGGTGG-3'<br>**R** 5'-CCAAGACATGGAGGAGCCAT-3'
let-7i-5p	**F** 5'-TGAGGTAGTAGTTTGTGCTGTT-3'<br>**R** 5'-GCGAGCACAGAATTAATACGAC-3'
miR-21-5p	**F** 5'-TTTTGTTTTGCTTGGGAGGA-3'<br>**R** 5′-AGCAGACAGTCAGGCAGGAT-3′
U6	**F** 5'-CTCGCTTCGGCAGCACA-3'<br>**R** 5'-AACGCTTCACGAATTTGCGT-3'

Gene	Primer Sequence
E-Cadherin	**F** 5'-ATTCTGATTCTGCTGCTCTTG-3'<br>**R** 5'-AGTCCTGGTCCTCTTCTCC-3'
N-Cadherin	**F** 5'-CCACGCCGAGCCCCAGTATC-3'<br>**R** 5'-CCCCCAGTCGTTCAGGTAATCA-3'

β-Actin	**F** 5’-CATGTACGTTGCTATCCAGGC-3’<br>**R** 5’-CTCCTTAATGTCACGCACGAT-3’
